# Preoperative Pain Control for a Femoral Neck Fracture Using Intra-Articular Multimodal Drug Injection

**DOI:** 10.3390/jcm15051762

**Published:** 2026-02-26

**Authors:** Konlawat Sabsuantang, Siraphat Ponghunsa, Jinnipa Arunothai, Vachirasorn Anannor, Atikun Natee, Paphon Sa-Ngasoongsong

**Affiliations:** 1 Chakri Naruebodindra Medical Institute, Faculty of Medicine Ramathibodi Hospital, Mahidol University, Samut Prakan 10540, Thailand; konlawat.sab@mahidol.ac.th (K.S.); vachirasorn.ana@mahidol.ac.th (V.A.); atikun.nat@mahidol.ac.th (A.N.); 2 Department of Orthopedics, Faculty of Medicine Ramathibodi Hospital, Mahidol University, Bangkok 10400, Thailand; siraphat.pon@mahidol.ac.th (S.P.); jinnipa.arn@mahidol.ac.th (J.A.)

**Keywords:** elderly hip fracture, intra-articular injection, pain management, morphine consumption, multimodal analgesia

## Abstract

**Background/Objectives:** Hip fractures among elderly patients are associated with significant morbidity and mortality. Delayed surgery is common and often results in inadequate pain control and increased opioid consumption, which may have adverse effects. This study evaluates the effectiveness of preoperative intra-articular injection of multimodal analgesics (IA MDI) for reducing pain caused by a displaced femoral neck fracture (FNF). **Methods:** A prospective randomized controlled trial was conducted using 18 geriatric patients with displaced FNFs scheduled for hip arthroplasty. The patients were randomized into two groups: IA MDI and control groups (*n* = 9 each). The IA MDI group was administered a preoperative intra-articular injection of ropivacaine, morphine, and adrenaline, in addition to standard oral and intravenous (IV) analgesics, while the control group was administered standard oral and IV analgesics alone. The primary outcome was the perioperative pain score assessed via the 10-point numerical rating scale (NRS). The secondary outcomes were morphine consumption, perioperative complications, length of hospital stay, and functional outcome. **Results:** During the first 24 h preoperative period after admission, the IA MDI group exhibited a significant reduction in the average NRS at all timepoints (*p* < 0.05 all) and in the median dosage of morphine consumption (0 mg vs. 6 mg, *p* = 0.033) compared to the control group. There was no significant difference between groups in terms of postoperative pain and complications, length of hospital stays, or functional outcomes (*p* > 0.05 all). **Conclusions:** Preoperative IA MDI significantly reduced pain intensity and opioid consumption during the preoperative 24 h window among elderly patients with FNFs without provoking a corresponding increase in observed complications in this pilot randomized controlled study. IA MDI is a feasible option and could be a useful adjunct for preoperative pain management for FNFs.

## 1. Introduction

Femoral neck fractures (FNFs) are common and critical injuries in the elderly population associated with a high 1-year mortality rate of up to 30% [1]. Acute pain originating from the fracture site significantly restricts patient mobility, resulting in prolonged immobilization and a cascade of life-threatening complications, including pressure ulcers, pneumonia, and urinary tract infections [2]. Though early surgical management within 48–72 h is the standard for reducing mortality and morbidity, surgery is often delayed for several reasons, such as limited operating-room availability, the need to wait for the reversal of an antithrombotic agent, unstable medical condition, and a requirement for preoperative consultation [3]. Crucially, this inadequate preoperative pain control is a major contributor to preoperative complications and makes both early mobilization and subsequent postoperative rehabilitation extremely challenging [4].

The current standard for preoperative pain management for FNFs involves systemic administration of analgesics such as opioids and NSAIDs. However, this approach is often insufficient, and it is associated with significant systemic side effects (e.g., confusion, nausea, and renal impairment), which are particularly harmful to elderly patients [5]. While regional anesthesia techniques like various types of nerve blocks have been developed to manage the complex innervation of pain-originating structures, including hip capsules, fracture ends, and the periosteum, and offer better localized relief, their use can be limited by the heterogeneity of resources and the availability of expertise across centers [6,7].

Intra-articular and peri-articular injections allow targeted pain control of hip structures and are more familiar and available options for most orthopedic surgeons. Multimodal analgesic regimens are beneficial for hip-fracture pain management, as they cover various mechanisms of pain and allow for lower doses of each drug, thereby reducing complications [8]. Peri-articular injection with multiple analgesic regimens during hip arthroplasty was recently proven to be effective in postoperative pain management, with no significant complications [9,10,11]. Hence, the application of a multimodal analgesic regimen administered intra-articularly prior to surgery could be a complementary, feasible method orthopedic surgeons can use to provide superior short-term analgesia and serve as an opioid-sparing strategy in preoperative pain management for elderly patients with FNFs. Moreover, this intra-articular injection can be administered quickly under fluoroscopic guidance at the time of admission, thereby reducing the need for specialized, time-consuming ultrasound guidance for peripheral nerve blocks. However, to the best of our knowledge, no randomized controlled trials evaluating the efficacy of preoperative intra-articular multimodal drug injection (IA MDI) for FNFs have been conducted.

We hypothesized that elderly patients with FNFs who were administered IA MDIs would have lower preoperative pain scores and consume less morphine than patients administered a standard multimodal analgesic regimen. Thus, we aimed to evaluate the effectiveness of preoperative IA MDI relative to standard intravenous and oral analgesic protocols in the preoperative period among elderly patients with FNFs in terms of pain score reduction, opioid consumption, and postoperative outcomes.

## 2. Materials and Methods

### 2.1. Study Design and Setting

This multi-center, single-blinded (assessor-blinded), randomized controlled trial was conducted between October 2023 and December 2025 at Ramathibodi Hospital, Faculty of Medicine Ramathibodi Hospital, Mahidol University, Bangkok, Thailand, and Chakri Naruebodindra Medical Institute, Faculty of Medicine Ramathibodi Hospital, Mahidol University, Samut Prakan, Thailand. The study protocol was approved by the Institutional Review Board prior to participant enrollment (COA No. MURA 2023/479). The trial was prospectively registered with the Thai Clinical Trials Registry on 17 September 2023 (Registration ID: TCTR20230918005), and has been reported in accordance with the CONSORT guidelines.

### 2.2. Participants and Eligibility Criteria

Patients presenting with a closed femoral neck fracture at Ramathibodi Hospital and Chakri Naruebodindra Medical Institute between October 2023 and June 2025 were screened for eligibility. According to the inclusion criteria, we included patients aged 60 years or older who sustained closed displaced femoral neck fractures caused by low-energy trauma and were scheduled for hemiarthroplasty. The exclusion criteria included having sustained multiple trauma or fractures; having a history of hip fractures or surgery; having a pathological fracture other than osteoporosis; pre-existing ipsilateral hip osteoarthritis; an injury onset greater than 72 h; being allergic to the study medications; poorly controlled diabetes mellitus (HbA1c > 8%); current systemic steroid use; having a local infection at the injection site; and severe cognitive impairment that could affect data collection.

### 2.3. Sample-Size Calculation

Sample-size estimation was performed using the N4 Studies application based on data from a pilot study of 10 elderly patients with femoral neck fractures, in which a mean pain score of 5.2 was reported. Clinically significant pain reduction was defined as a decrease of at least 1.3 points [12], resulting in an expected mean pain score of 3.9 in the treatment group. With a power of 0.8 and a significance level of 0.05, and allowing for a 20% dropout rate, a total sample size of 18 patients (9 per group) was required.

### 2.4. Randomization

Patients were randomly assigned in a 1:1 ratio to either the IA MDI group (*n* = 9) or the control group (*n* = 9) using a computer-generated blocked randomization sequence generated via STATA software version 16.0 (StataCorp, College Station, TX, USA). Then, the randomization sequence was prepared in sequentially numbered, opaque, sealed envelopes. After informed consent was obtained and the participants were enrolled in the emergency department (ED), a research assistant, who was not involved in data collection, opened the sealed envelopes. The patients in the control group were then admitted into the orthopedic ward, while those in the IA MDI group were sent to the operating room to undergo the IA MDI procedure before ward admission.

### 2.5. Intervention

#### 2.5.1. Intra-Articular Multimodal Drug Injection

**Figure 1:**
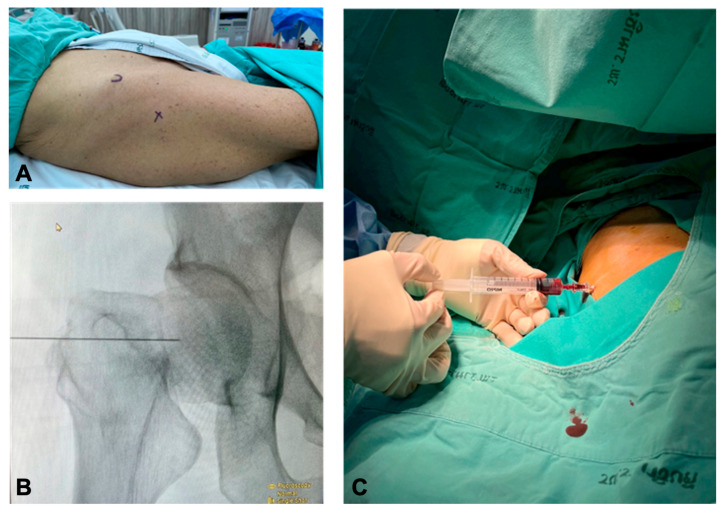
Intra-articular multimodal drug injection technique. (**A**). Lateral-based hip joint injection approach. (**B**). Needle insertion anterior to greater trochanter and checked with fluoroscope. (**C**). Negative aspiration after bone contact to check intra-articular blood.

Each patient was placed in a supine position without anesthesia. The injection site was prepared using a sterile technique. The injection solution was prepared by an operating room nurse; it was made up to a total volume of 10 mL, containing 50 mg of ropivacaine, 10 mg of morphine, and 300 mcg of adrenaline diluted with normal saline. The solution was administered using a 25-gauge spinal needle. The needle entry point was located just anterior to the greater trochanter and inserted perpendicular to the skin in the coronal plane using a lateral-based approach [13,14], with modifications made based on the assumption of femoral proximal migration and external rotation after femoral neck fractures. After bone contact was made, negative aspiration was performed to exclude blood or synovial fluid, and fluoroscopic confirmation was used to verify intra-articular placement before injection (Figure 1).

#### 2.5.2. Control Intervention

The patients in the control group were not administered an intra-articular injection, and they were managed with the same standard preoperative pain control protocol, including intravenous and oral analgesics.

### 2.6. Pain Management Protocol

Baseline pain intensity was assessed upon presentation to the emergency department prior to randomization to maintain assessor blinding. All patients were intravenously administered 3 mg of morphine sulphate at ED. The research assistant, who did not know the randomization sequence, reassessed pain intensity after injection in the IA MDI group and after admission in the control group. Pain scores were subsequently recorded every 8 h using a 10-point numerical rating scale (NRS). Apart from IA MDI, both groups were subjected to identical analgesic regimens, including intravenous morphine sulfate (3 mg every 4 h as needed for NRS > 4), with rescue doses of 1.5 mg if pain persisted after 60 min. Oral acetaminophen (500 mg every 6 h) was administered routinely. Opioid-related adverse effects were managed with intravenous metoclopramide (10 mg) as required. This protocol was followed until surgery.

### 2.7. Outcome Measures and Data Collection

Demographic data, including age, sex, body mass index (BMI), and comorbidities, were collected preoperatively by a research assistant. The primary outcome was assessed using the 10-point numerical rating scale (NRS) after intervention (in the IA MDI group) or after admission (in the control group) every 8 h during the first 24 h period before definitive surgical treatment. Due to the time of the IA MDI procedure, the first NRS pain score assessment for the IA MDI group (0 h timepoint) was delayed by approximately 1 h relative to the assessment for the control group. However, the later NRS pain score timepoints (8 h, 16 h, and 24 h) were all scheduled from the time of admission for both groups.

Total morphine consumption was calculated as the cumulative dose administered preoperatively from admission to surgery. Opioid-related adverse effects, including oversedation and opioid-induced respiratory depression, were recorded. Injection-related complications, such as local bleeding or ecchymoses, inadvertent extra-articular placement, and neurovascular injury, were also recorded. Perioperative complications (local infections, delirium, deep-vein thrombosis, coronary artery disease, gastrointestinal bleeding, and nausea/vomiting), length of stay, and ambulatory level at discharge were identified through a review of medical records during hospitalization and recorded as the number of patients with the corresponding condition. Delirium was assessed using the confusion assessment method. Periprosthetic joint infection, ambulatory level, and readmission were assessed at follow-up visits for up to 6 months after surgery.

Ambulatory level was classified into three levels: non-ambulatory (bed-bound and wheelchair-dependent), ambulatory with a gait aid, and ambulatory without a gait aid. Return to pre-fracture ambulatory level (RPAL) was defined as regaining the original level of ambulation from before suffering a fracture, as assessed 6 months after surgery.

### 2.8. Statistical Analysis

Statistical analysis was performed using the Statistical Package for the Social Sciences version 18 (SPSS Inc., Chicago, IL, USA). Data distribution was assessed using the Kolmogorov–Smirnov test. Continuous variables were analyzed using Student’s *t*-test, a Mann–Whitney U test, or Repeated-Measures ANOVA as appropriate, while categorical variables were compared using the chi-square or Fisher’s exact test. A *p*-value of ≤0.05 was considered statistically significant.

## 3. Results

### 3.1. Participant Flow and Baseline Characteristics

**Figure 2:**
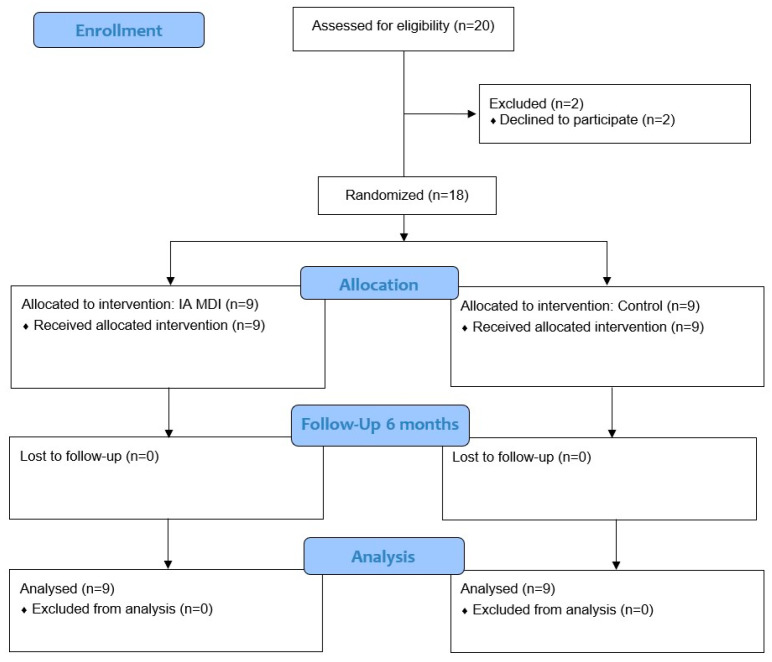
CONSORT flow diagram of participant enrollment, allocation, follow-up, and analysis.

A total of 18 patients were enrolled and randomized into the IA MDI group and the control group, with 9 patients in each group. No patients were lost to follow-up, and all randomized participants were included in the final analysis. The flow of participants throughout the study is shown in Figure 2.

The baseline demographic and clinical characteristics of the two groups are summarized in Table 1. There were no significant differences between the IA MDI and control groups with respect to age, sex, BMI, fracture side, comorbidities, or baseline pain scores upon presentation to the emergency department (*p* > 0.05 for all).

### 3.2. Pain Outcomes

**Figure 3:**
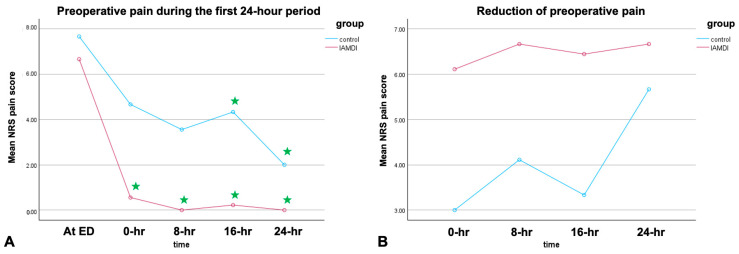
Pain outcomes during the preoperative period: (**A**) preoperative pain score during the first 24 h period, and (**B**) the difference in pain scores between the initial pain score recorded in the emergency department and that recorded at later timepoints. ★, significant difference, with a *p*-value < 0.05, determined via post hoc analysis.

Regarding the NRS pain outcomes, we found that the IA MDI group had a significantly lower average pain score in the first 24 h after injection relative to the control group (*p* = 0.008) (Table 2). Post hoc analysis showed that the NRS pain score after IA MDI was significantly lower than the initial score recorded at the emergency department at all timepoints (*p* < 0.05 all, Figure 3). The reduction in preoperative pain in the IA MDI group was non-statistically greater than that in the control group (0.075). However, the postoperative pain scores were not significantly different between groups (*p* = 0.275) (Table 2 and Figure 3).

### 3.3. Morphine Consumption and Complications

Concerning morphine consumption, the IA MDI also significantly decreased preoperative morphine consumption during first 24 h relative to the control group (*p* = 0.033) (Table 3). The mean difference (95% confidence interval) between the IA MDI and control groups was −4.3 (−7.8 to −0.9).

Both groups showed non-significant differences in perioperative complications and length of hospital stay (*p* > 0.05 all). There was one case from each group (11.1%) that featured preoperative delirium. There were no periprosthetic joint infections over the 6-month follow-up period.

### 3.4. Functional Outcomes

The functional outcomes analyzed are presented in Table 2. There were no significant differences in ambulatory level at discharge and return to pre-fracture ambulatory level between groups.

## 4. Discussion

Adequate pain control is one of the most important factors for managing elderly hip fracture patients. Recently, a randomized control trial comparing a fascia iliaca compartment block (FICB) with intra-articular hip injection (IAHI) using pure ropivacaine found that the intervention for the IAHI group reduced pain to a greater degree than that for the FICB group, with no serious complications [15]. In this study, we aimed to evaluate the effectiveness of IA MDI without NSAIDs for preoperative pain control in elderly individuals with displaced femoral neck fractures in comparison with systemic analgesics.

The results of this study are reported through an assessment of NRS, morphine consumption, and complications. A comparison of the NRS pain score of 10-point NRS and the amount of morphine used preoperatively indicated significant pain reduction in the IA MDI group compared to the control group during the first preoperative 24 h (Table 2 and Table 3 and Figure 3). Post hoc analysis demonstrated that the NRS pain score in the IA MDI group was significantly lower than the initial score noted at the emergency department at all recorded timepoints (*p* < 0.05 all, Figure 3), indicating a significant time–group interaction. This result could be due to the effect of IA-MDI on the fracture site and inflamed hip capsule. Moreover, IA MDI can also reduce the median dose of preoperative morphine consumption in the first 24 h (0 mg vs. 6 mg, *p* = 0.033). Our centers aim to operate on elderly patients with hip fractures in accordance with an early hip fast-track surgery protocol; therefore, the data on the IA MDI effect after 24 h might be lacking. No serious complications were detected in the IA MDI group. The results indicate that IA MDI is beneficial for preoperative pain management for at least 24 h and safe for elderly femoral neck fracture patients.

A duration of effect of at least 24 h significantly exceeds the known duration of action of the drugs used in the regimen. This prolonged effect might be explained by the minimal systemic absorption from the addition of adrenaline and effective intra-articular containment of drugs from the intact hip capsule in low-energy trauma. Moreover, early aggressive pain management may improve psychological well-being and prevent subsequent severe pain, in line with the concepts of preemptive analgesia and the prevention of central sensitization [16]. The benefit of IA MDI hip injection in our study aligns with a study by Aprato et al. who used a 100 mg ropivacaine solution for IAHI, reporting better pain control for up to 48 h compared to an FICB [15].

To the best of our knowledge, our study is the first to investigate the effect of IA MDI for preoperative pain management on FNFs. In contrast to a previous study on pure ropivacaine IA injection [15], we utilized a multimodal drug injection in order to reduce the dosage (50 mg of ropivacaine) and potential complications associated with high-dose single-agent regimens. While ropivacaine is the least chondrotoxic local anesthetic, the use of high doses and concentrations remains a concern [17]. Morphine sulphate was included due to its minimal systemic absorption, lack of chondrotoxicity, and the known presence of opioid receptors in peripheral tissues [18,19,20]. Adrenaline was added to further minimize systemic absorption. Notably, NSAIDs were not used, as chronic kidney disease is common in elderly patient populations. Consequently, we believe that our multimodal drug regimen is both feasible and beneficial for this specific group of patients. Regarding the IAHI method, we used a fluoroscopy-assisted, modified lateral approach. This approach was adapted based on common fracture displacement patterns and chosen because it is familiar to most orthopedic surgeons. The combination of the chosen regimen and technique enhances the generalizability of our findings.

Our study also has some limitations. First, although this study was a randomized controlled trial without dropout, our sample size was small, and there was a potential confounder due to the near-imbalance of the side distribution (Table 1), resulting in limited power for detecting postoperative complications. Moreover, due to the single-blinded design, the intervention in the IA MDI group usually took approximately 1 h, resulting in an approximately 1 h delay for the initial NRS collection relative to the control group, and this issue could be considered to have introduced a potential performance bias via non-equivalent early measurement timing. Therefore, in terms of using a repeated-measures ANOVA assumption in a small cohort and the effect of IA MDI on the postoperative outcomes, functional recovery, or uncommon adverse events due to the pilot nature of this study (6-month follow-up period), the results should be cautiously interpreted. Hence, further larger trials with an improved methodology comparing IA MDI with other preoperative regional anesthesia techniques (such as FICB) are needed to emphasize the role of IA MDI in preoperative pain and delirium management and identify the postoperative outcomes and complications related to IA MDI, including infections associated with the infiltration technique employed. Second, while the ultrasound-guided hip injection has been proven to have higher accuracy for hip joint injection [21], we used a combined landmark-based approach and confirmation with blood aspiration and a fluoroscope due to orthopedic surgeons’ familiarity with this technique. Therefore, our results might not be directly applicable to cases involving ultrasound-guided hip injection. Furthermore, the implementation of this IA MDI for FNFs in general practice requires careful consideration of the necessary infrastructure, specifically fluoroscopy and operating-room access. Lastly, as early fast-track surgery is required for patients with femoral neck fractures, the time allotted to collecting data was limited. Future studies might reveal differences in NRS scores between groups 24 h after injection if data can be adequately collected for more than 24 h. The results of this study prove the advantages of IA MDI for elderly patients with FNFs in terms of its efficacy as an adjunct to preoperative multimodal analgesic practices (systemic acetaminophen and low-dose opioid administration, with or without a peripheral nerve block), especially in those with severe pain at the initial presentation and those with a contraindication for systemic analgesic medications. However, IA MDI also has disadvantages due to the requirement for related resources (fluoroscopy and operating-room access) and expertise in intra-articular hip injection.

## 5. Conclusions

In conclusion, intra-articular multimodal drug injection (IA MDI) was found to be associated with reduced preoperative pain and morphine consumption during the first 24 h after injection among elderly patients with femoral neck fractures in this small, randomized study. Moreover, no increases in complications were observed in this limited sample. Larger and more adequately powered trials are required to confirm safety and efficacy. IA MDI may serve as a useful adjunct for managing preoperative pain and reducing systemic opioid use in this patient population.

## Figures and Tables

**Table 1:** Baseline demographic and clinical characteristics of the participants.

	IA MDI (*n* = 9)	Control (*n* = 9)	*p*-Value
Age (year)	78.2 ± 5.6	79.8 ± 7.4	0.62
Gender (female, %)	8 (88.9%)	8 (88.9%)	1.00
BMI (kg/m^2^)	21.2 ± 2.6	20.8 ± 3.6	0.77
Side (left, %)	2 (22.2%)	6 (66.7%)	0.06
Comorbidity (%)			
Essential hypertension	7 (77.8%)	8 (88.9%)	0.53
Diabetes	2 (22.2%)	4 (44.4%)	0.32
Initial NRS pain score at ED	6.7 ± 2.8	7.7 ± 2.2	0.41

IA MDI, intra-articular multimodal drug injection; ED, emergency department; NRS, 10-point numerical rating scale.

**Table 2:** NRS Pain score.

	IA MDI (*n* = 9)	Control (*n* = 9)	∆ (95% CI)	*p*-Value ^a^
Preoperative pain (NRS)				0.008 *
At ED	6.7 ± 2.8	7.7 ± 2.2	−1.0 (−3.5 to 1.5)	
0 h	0.6 ± 1.7	4.7 ± 3.9	−4.1 (−7.2 to −1.0)	
8 h	0.0 ± 0.0	3.6 ± 4.2	−3.6 (−6.8 to −0.3)	
16 h	0.2 ± 0.7	4.3 ± 3.9	−4.1 (−7.2 to −1.1)	
24 h	0.0 ± 0.0	2.0 ± 2.7	−2.0 (−4.0 to 0.0)	
Reduction in preoperative pain (NRS) ^b^				0.075
0 h	6.1 ± 3.9	3.0 ± 3.3	3.1 (−0.5 to 6.7)	
8 h	6.7 ± 2.8	4.1 ± 3.3	2.6 (−0.5 to 5.6)	
16 h	6.4 ± 2.6	3.3 ± 2.6	3.1 (0.5 to 5.7)	
24 h	6.7 ± 2.8	5.7 ± 3.2	1.0 (−2.0 to 4.0)	
Postoperative pain (NRS)				0.275
0 h	2.0 ± 3.5	2.8 ± 3.9	−0.8 (−4.5 to 2.9)	
24 h	1.8 ± 3.0	1.9 ± 2.7	−0.1 (−3.0 to 2.7)	
48 h	0.4 ± 1.3	1.4 ± 2.3	−1.0 (−2.9 to 0.9)	
72 h	0.3 ± 1.0	1.2 ± 2.7	−0.9 (−3.0 to 1.3)	

∆, mean difference between the IA MDI and control groups; CI, confidence interval; ^a^, *p*-value calculated via repeated-measures ANOVA; ^b^, calculated from the difference between pain at ED and pain at the recorded timepoint; ED, emergency department; NRS, 10-point numerical pain scale. *, statistically significant, with *p* < 0.05.

**Table 3:** Comparison of morphine consumption, complications, and functional outcomes.

	IA MDI (*n* = 9)	Control (*n* = 9)	*p*-Value
Morphine dose (mg) ^a^			
Preoperative (24 h)	0 (0–3)	6 (2.25–9)	0.033 *
Complications (%) ^b^			
Local infection	0	0	-
Delirium	1 (11.1)	1 (11.1)	1.00
Deep-vein thrombosis	0 (0.0)	0 (0.0)	-
Coronary artery disease	0 (0.0)	0 (0.0)	-
Gastrointestinal bleeding	0 (0.0)	0 (0.0)	-
Length of stay (days) ^c^	7.2 ± 1.9	8.6 ± 3.4	0.32
Ambulatory at discharge (%)	6 (66.7)	6 (66.7)	1.00
Periprosthetic joint infection (%)	0 (0.0)	0 (0.0)	-
RPAL at 6 months (%)	66.7	66.7	1.00

^a^, value presented as median (interquartile range) and calculated using a Mann–Whitney U test. ^b^, value presented as number of cases (percentage) and calculated using a Fisher Exact test. ^c^, value presented as mean ± standard deviation and calculated using an unpaired *t*-test. RPAL, return to preambulatory level. *, significant difference with *p* < 0.05.

## Data Availability

The data presented in this study are available on request from the corresponding author due to participant privacy concerns.

## Abbreviations

The following abbreviations are used in this manuscript:

FNF	Femoral neck fracture
IA MDI	Intra-articular multimodal drug injection
ED	Emergency department
NRS	Numerical rating scale
BMI	Body mass index
RPAL	Return to pre-fracture ambulatory level
FICB	Fascia iliaca compartment block
IAHI	Intra-articular hip injection
